# Association between voltage-gated sodium channel gene polymorphisms and chronic oxaliplatin-induced peripheral neuropathy in Japanese patients with colorectal cancer

**DOI:** 10.1186/s40780-026-00601-2

**Published:** 2026-06-19

**Authors:** Masato Matsuura, Ayumu Nagamine, Takashi Masuno, Akira Tabei, Teruko Nasaka, Hiromi Nishiba, Maho Koike, Yuta Takahashi, Kyoko Obayashi

**Affiliations:** 1 Faculty of Pharmacy, Takasaki University of Health and Welfare, 60 Nakaorui-machi, Takasaki, Gunma 370-0033 Japan; 2 Department of Pharmacy, Gunma Saiseikai Maebashi Hospital, 564-1 Kamishinden-machi, Maebashi, Gunma 371-0821 Japan; 3 Department of Pharmacy, Fujioka General Hospital, 813-1 Nakakurisu, Fujioka, Gunma 375-0015 Japan; 4 Department of Pharmacy, NHO Takasaki General Medical Center, 36-36 Takamatsu-cho, Takasaki, Gunma 370-0829 Japan; 5 Department of Pharmacy, Isesaki Municipal Hospital, 12-1 Tsunatori- Honmachi, Isesaki, Gunma 372-0817 Japan; 6 Department of Pharmacy, JCHO Gunma Chuo Hospital, 1-7-13 Koun-cho, Maebashi, Gunma 371-0025 Japan; 7 Education Center for Clinical Pharmacy, Faculty of Pharmacy, Takasaki University of Health and Welfare, 60 Nakaorui-machi, Takasaki, Gunma 370-0033 Japan

**Keywords:** Oxaliplatin, Peripheral neuropathy, Sodium channel, SCN, Polymorphisms, Pharmacogenetics, Cancer

## Abstract

**Background:**

Polymorphisms in voltage-gated sodium channel (SCN) genes have been implicated in oxaliplatin-induced peripheral neuropathy (OXAIPN). However, their association with chronic OXAIPN in Japanese patients remains unclear. This study investigated the association between SCN gene polymorphisms and patient-reported chronic OXAIPN outcomes in Japanese patients with colorectal cancer.

**Methods:**

Seventy-six Japanese patients with colorectal cancer who received oxaliplatin-containing adjuvant chemotherapy were included. Seven polymorphisms, namely *SCN4A* rs2302237, *SCN5A* rs11720524, *SCN9A* rs6746030 and rs6754031, *SCN10A* rs6800541 and rs12632942, and *GSTP1* rs1695, were analyzed. Chronic OXAIPN was assessed using the Functional Assessment of Cancer Therapy/Gynecologic Oncology Group–Neurotoxicity (FACT/GOG-Ntx) score as the primary endpoint, while symptom severity assessed using the Patient-Reported Outcomes version of the Common Terminology Criteria for Adverse Events (PRO-CTCAE) was evaluated as a key secondary endpoint. Assessments were conducted at treatment completion (0 M) and 6 months after treatment completion (6 M).

**Results:**

The mean age of the patients was 64.6 ± 9.9 years, and 50 patients (65.8%) were male. The mean cumulative oxaliplatin dose was 792 ± 184 mg/m². In multivariate analyses, carriers of the *SCN4A* rs2302237 variant allele had lower FACT/GOG-Ntx scores than non-carriers at both 0 M (B = − 4.385, 95% confidence interval [CI]: −7.938 to − 0.831, *p* = 0.016) and 6 M (B = − 5.150, 95% CI: −8.975 to − 1.326, *p* = 0.009), indicating a lower patient-reported neuropathy-related symptom burden. In contrast, carriers of the *SCN10A* rs6800541 variant allele had higher FACT/GOG-Ntx scores at 0 M (B = 4.564, 95% CI: 0.393 to 8.735, *p* = 0.032); however, no similar association was observed at 6 M. PRO-CTCAE severity analyses showed similar trends for *SCN4A* rs2302237 and *SCN10A* rs6800541.

**Conclusions:**

In this exploratory study of Japanese patients with colorectal cancer, *SCN4A* rs2302237 was consistently associated with a lower neuropathy-related symptom burden at both 0 M and 6 M. These findings suggest that *SCN4A* rs2302237 may be a candidate marker associated with patient-reported chronic OXAIPN symptom burden; however, larger validation studies are needed to confirm its clinical significance.

**Supplementary Information:**

The online version contains supplementary material available at https://doi.org/10.1186/s40780-026-00601-2.

## Background

Colorectal cancer is one of the most common cancers worldwide, making it a major contributor to cancer-related mortality. Oxaliplatin-based regimens are widely used as standard adjuvant chemotherapy after curative resection to reduce the risk of recurrence [1, 2]. However, oxaliplatin treatment is frequently complicated by oxaliplatin-induced peripheral neuropathy (OXAIPN). OXAIPN is characterized by both acute neuropathy, which is transient and includes symptoms such as cold hypersensitivity and perioral paresthesia, and chronic neuropathy, which is marked by persistent sensory impairment and numbness in the extremities [3]. Chronic OXAIPN is of particular clinical importance because it can persist after the completion of chemotherapy, impair daily functioning, and lead to long-term deterioration in quality of life (QOL) [4, 5]. Several clinical factors have been reported to be associated with OXAIPN, including cumulative oxaliplatin dose, number of treatment cycles, age, body mass index (BMI), diabetes mellitus, sex, anemia, alcohol use, pre-existing neuropathy, and concomitant medications [6, 7]. Nevertheless, substantial interindividual variability exists in the occurrence and severity of OXAIPN, which cannot be completely explained by clinical factors alone.

Genetic factors may contribute to the risk of developing platinum-based chemotherapy-induced peripheral neuropathy (CIPN). Glutathione S-transferases are enzymes that play an important role in detoxification processes, while polymorphisms in *GSTP1* have been reported to be associated with platinum-induced peripheral neurotoxicity in multiple studies [8–10]. In addition, single-nucleotide polymorphisms (SNPs) in genes related to DNA repair, membrane transport, drug metabolism, and ion channels have been investigated for their potential associations with CIPN [11]; however, the number of such studies remains limited, and the reproducibility and consistency of their findings have not been sufficiently validated. Under these circumstances, recent studies have reported that SNPs in voltage-gated sodium channel (*SCN*) genes may be involved in the development of OXAIPN [12]. The *SCN* gene family (*SCN1A*–*SCN11A*) encodes the α-subunits of voltage-gated sodium channels (Nav), which are widely expressed in excitable tissues, including the nervous system, heart, and skeletal muscle, and play a critical role in the initiation and propagation of action potentials [13]. Notably, certain Nav subtypes that are highly expressed in nociceptive neurons, such as Nav1.7 and Nav1.8, are known to be involved in pain perception and signal amplification [14]. Oxaliplatin is thought to contribute to the development of OXAIPN-specific symptoms, such as cold hypersensitivity and neuropathic pain, by altering Nav inactivation kinetics and inducing hyperexcitability of sensory neurons [15].

To date, associations between OXAIPN and genetic polymorphisms in several *SCN* genes, including *SCN4A* (Nav1.4), *SCN5A* (Nav1.5), *SCN9A* (Nav1.7), and *SCN10A* (Nav1.8), have been investigated. Argyriou et al. [16] reported, in a prospective multicenter study mainly involving European patients, that *SCN4A* rs2302237 was associated with the development of OXAIPN. In addition, Sereno et al. [17] reported that Spanish patients with gastrointestinal cancer carrying the *SCN9A* rs6746030 polymorphism exhibited a significantly lower incidence of OXAIPN than those without this polymorphism. Furthermore, a study by Palugulla et al. [18] involving South Indian patients with gastrointestinal cancer showed that polymorphisms in *SCN4A* rs2302237, *SCN9A* rs6746030, and *SCN10A* rs12632942 were associated with the development and severity of chronic OXAIPN, whereas *SCN5A* rs11720524 showed no such association. Although accumulating evidence suggests an association between *SCN* gene polymorphisms and the risk of OXAIPN, these findings have predominantly been derived from European and South Asian populations. Because allele frequencies of *SCN* gene polymorphisms may differ across ethnic backgrounds, evidence from East Asian populations is necessary to clarify the generalizability and clinical relevance of previously reported associations. To date, no studies have specifically examined the association between *SCN* gene polymorphisms and chronic OXAIPN in East Asian populations, particularly in Japanese patients. Therefore, we aimed to investigate the association between *SCN* gene polymorphisms and chronic OXAIPN in Japanese patients with colorectal cancer.

## Methods

### Patients

This multicenter prospective observational study was conducted at five institutions in Japan (Gunma Saiseikai Maebashi Hospital, Fujioka General Hospital, NHO Takasaki General Medical Center, Isesaki Municipal Hospital, and JCHO Gunma Chuo Hospital). The study protocol was approved by the ethics committees of all participating institutions, and written informed consent was obtained from all participants before enrollment.

Patients with colorectal cancer who received oxaliplatin-containing adjuvant chemotherapy, either CAPOX or mFOLFOX6, between January 2023 and January 2025 were enrolled. The choice of chemotherapy regimen was determined by the attending physicians at each institution, following routine clinical practice. In the CAPOX regimen (oxaliplatin plus capecitabine), oxaliplatin was administered at a dose of 130 mg/m² every 3 weeks for up to eight cycles. In the mFOLFOX6 regimen (oxaliplatin plus leucovorin and fluorouracil), oxaliplatin was administered intravenously at a dose of 85 mg/m² every 2 weeks for up to 12 cycles. The exclusion criteria were as follows: patients younger than 18 years of age, patients deemed by physicians to have difficulty in appropriately responding to questionnaires or assessments, patients who were lost to follow-up or had substantial missing data during the observation period, and patients who had evident peripheral neuropathy before the initiation of chemotherapy.

### Clinical data collection

Clinical data, including age, sex, height, weight, body surface area, comorbidities including diabetes mellitus, colorectal cancer stage according to the Union for International Cancer Control TNM classification (8th edition), treatment regimen, number of oxaliplatin-containing cycles, cumulative oxaliplatin dose, and concomitant medications were collected from electronic medical records.

### Assessment of peripheral neuropathy

Peripheral neuropathy was primarily evaluated using the Japanese version of the Functional Assessment of Cancer Therapy/Gynecologic Oncology Group–Neurotoxicity (FACT/GOG-Ntx) subscale. This instrument comprises 11 items assessing sensory, motor, and auditory neuropathy-related symptoms, each rated on a five-point scale (0 = not at all to 4 = quite a bit), yielding a total score ranging from 0 to 44. In the present study, raw FACT/GOG-Ntx item scores were summed without reverse scoring; therefore, higher scores indicate greater neuropathy-related symptom burden. The FACT/GOG-Ntx score was defined as the primary outcome because it quantitatively captures patient-reported neuropathy-related symptoms and their impact on daily functioning. Patient-reported peripheral neuropathy symptoms were additionally assessed using the Patient-Reported Outcomes version of the Common Terminology Criteria for Adverse Events (PRO-CTCAE), version 1.0 (Japanese version). Patients were asked to report the severity of numbness and tingling in the hands and feet. Each item was rated on a five-point scale (severity: none to very severe). Clinically meaningful peripheral neuropathy was defined as symptom severity of moderate or greater [19]. Among the PRO-CTCAE items, symptom severity was treated as a principal secondary outcome to improve readability and avoid giving equal interpretive weight to multiple correlated outcomes. Patient-reported assessments using FACT/GOG-Ntx and PRO-CTCAE were conducted at treatment completion (0 M) and repeated 6 months after treatment completion (6 M). In this study, 0 M was defined as the end of the final oxaliplatin-containing treatment cycle for the assessment of OXAIPN. Accordingly, when oxaliplatin-containing treatment was discontinued before completion of the planned regimen because of adverse events, patient preference, or other clinical reasons, or when fluoropyrimidine-based treatment without oxaliplatin was continued after oxaliplatin discontinuation, the final oxaliplatin administration cycle was regarded as 0 M.

### Genetic analysis

A total of seven SNPs in the *SCN* genes (*SCN4A*, *SCN5A*, *SCN9A*, and *SCN10A*) and the *GSTP1* gene (*SCN4A* rs2302237, *SCN5A* rs11720524, *SCN9A* rs6746030 and rs6754031, *SCN10A* rs6800541 and rs12632942, and *GSTP1* rs1695) were analyzed in this study. These SNPs were selected because previous studies had investigated their associations with OXAIPN or platinum-related neurotoxicity, and because *SCN* genes are biologically plausible candidates for oxaliplatin-related sensory symptoms [16, 17, 18]. *GSTP1* rs1695 was included as a comparative candidate polymorphism because *GSTP1* is involved in platinum detoxification and has been examined in relation to platinum-induced neurotoxicity. After written informed consent was obtained, residual whole-blood samples collected during routine clinical care after initiation of treatment were used for genetic analysis. Genomic DNA was extracted from whole blood samples using the QIAamp^®^ Blood Kit (Qiagen, Valencia, CA, USA). Genotyping was performed using TaqMan genotyping assays (Applied Biosystems, Foster City, CA, USA). TaqMan primers and probe sets designed for each SNP allele were included in the assay kits. The following assay IDs were used: *SCN4A* rs2302237, C__15757352_20; *SCN5A* rs11720524, C__30666704_10; *SCN9A* rs6746030, C__29330435_10; *SCN9A* rs6754031, C__29108389_20; *SCN10A* rs6800541, C__30572611_10; *SCN10A* rs12632942, C__31683397_10; and *GSTP1* rs1695, C__3237198_20. Genotyping results for each SNP were categorized by comparing carriers of the variant allele with non-carriers.

### Statistical analysis

Genetic polymorphisms were analyzed using a dominant model, comparing variant allele carriers with non-carriers. The primary outcome was the FACT/GOG-Ntx score. For FACT/GOG-Ntx scores, comparisons between genotype groups were conducted using the Mann–Whitney U test in univariate analyses, while multiple linear regression analysis was applied for multivariate analyses. For PRO-CTCAE severity, comparisons between genotype groups were conducted using Pearson’s chi-square test or Fisher’s exact test in univariate analyses, while logistic regression analysis was applied for multivariate analyses. Diabetes mellitus and cumulative oxaliplatin dose were selected a priori as adjustment factors because they have been reported as clinically important risk factors for OXAIPN. Given the limited sample size, the number of covariates was intentionally restricted to avoid model instability and overadjustment. To further address the possibility of false-positive findings or confounding by clinical background, exploratory comparisons of known OXAIPN-related clinical factors were performed between variant allele carriers and non-carriers. These comparisons focused particularly on *SCN4A* rs2302237, which showed the most consistent association with patient-reported outcomes, and included age, sex, BMI, cumulative oxaliplatin dose, and number of oxaliplatin-containing cycles. The Hardy–Weinberg equilibrium (HWE) for the genotype distribution of each SNP was assessed using the chi-square test. This study was exploratory and hypothesis-generating. Statistical significance was set at *p* < 0.05. All statistical analyses were performed using SPSS software version 26.0 (SPSS Inc., Chicago, IL, USA).

## Results

### Patient characteristics

This study enrolled 76 Japanese patients with colorectal cancer who received oxaliplatin-containing adjuvant chemotherapy. Patient characteristics at treatment initiation and treatment exposure are summarized in Table 1. The mean age was 64.6 ± 9.9 years, and 50 patients (65.8%) were men. The mean body surface area was 1.64 ± 0.20 m², the median number of oxaliplatin-containing cycles was 8 (interquartile range, 6–8), and the mean cumulative oxaliplatin dose was 792 ± 184 mg/m². No patients received concomitant medications intended to treat CIPN-related symptoms, including duloxetine, venlafaxine, or gabapentinoids, during the evaluation period. The genotype distributions of the genetic polymorphisms are presented in Table 2. No deviation from the HWE was observed in the analyzed variants.

**Table 1:** Patient characteristics at treatment initiation and treatment exposure

Characteristic	*n* = 76
Age, years	64.6 ± 9.9
Sex, male	50 (65.8)
BSA, m²	1.64 ± 0.20
Comorbidities	
Diabetes mellitus	7 (9.2)
CRC stage	
Stage Ⅱ	7 (9.2)
Stage Ⅲ	66 (86.8)
Stage Ⅳ	3 (4.0)
Treatment regimen	
CAPOX	75 (98.7)
mFOLFOX6	1 (1.3)
Number of oxaliplatin-containing cycles	8 (6–8)
Cumulative oxaliplatin dose, mg/m²	792 ± 184

Data are presented as the number (percentage), mean ± standard deviation, or median (interquartile range), as appropriate

Abbreviations: BSA, body surface area; CRC, colorectal cancer; IQR, interquartile range

**Table 2:** Genotype distributions of the selected SNPs

Gene/SNP	Wild-type homozygous	Mutant heterozygous	Mutant homozygous
*SCN4A* rs2302237	CC	CT	TT
	29 (38.1)	37 (48.7)	10 (13.2)
*SCN5A* rs11720524	CC	CG	GG
	55 (72.4)	21 (27.6)	0 (0.0)
*SCN9A* rs6746030	GG	GA	AA
	69 (90.8)	7 (9.2)	0 (0.0)
*SCN9A* rs6754031	TT	TG	GG
	44 (57.9)	32 (42.1)	0 (0.0)
*SCN10A* rs6800541	TT	TC	CC
	56 (73.7)	15 (19.7)	5 (6.6)
*SCN10A* rs12632942	AA	AG	GG
	23 (30.3)	35 (46.0)	18 (23.7)
*GSTP1* rs1695	AA	AG	GG
	63 (82.9)	12 (15.8)	1 (1.3)

Data are presented as the number (percentage)

Abbreviations: SNP, single nucleotide polymorphism

### Patient-reported assessments of chronic OXAIPN

The patient-reported assessments of chronic OXAIPN are summarized in Table 3. The mean FACT/GOG-Ntx score decreased from 12.42 ± 8.20 at 0 M to 10.57 ± 8.81 at 6 M, suggesting an overall improvement in neuropathy-related symptom burden after treatment completion. For PRO-CTCAE severity, 63.1% of patients reported moderate or greater peripheral neuropathy at 0 M, whereas this proportion decreased to 38.2% at 6 M.

**Table 3:** Patient-reported assessments of peripheral neuropathy

Variable	At treatment completion (0 M)	6 months after treatment completion (6 M)
FACT/GOG-Ntx score	12.4 ± 8.2	10.6 ± 8.8
PRO-CTCAE “severity”		
None	4 (5.3)	16 (21.0)
Mild	24 (31.6)	31 (40.8)
Moderate	24 (31.6)	17 (22.4)
Severe	18 (23.6)	11 (14.5)
Very severe	6 (7.9)	1 (1.3)

Data are presented as the number (percentage) or mean ± standard deviation

Abbreviations: FACT/GOG-Ntx, Functional Assessment of Cancer Therapy/Gynecologic Oncology Group–Neurotoxicity; PRO-CTCAE, Patient-Reported Outcomes version of the Common Terminology Criteria for Adverse Events

### Association between SNPs and chronic OXAIPN

Carriers of the *SCN4A* rs2302237 and *GSTP1* rs1695 variant alleles had lower FACT/GOG-Ntx scores than non-carriers at both 0 M and 6 M in the univariate analyses (Table 4). These associations remained significant in the multivariate analyses (Table 5). In contrast, carriers of the *SCN10A* rs6800541 variant allele had higher FACT/GOG-Ntx scores at 0 M, whereas a similar association was not observed at 6 M. As an additional exploratory analysis, clinical risk factors were compared between *SCN4A* rs2302237 variant allele carriers and non-carriers (Additional file 1, Table S1). No apparent differences in age, sex, BMI, diabetes mellitus, cumulative oxaliplatin dose, or number of oxaliplatin-containing cycles were observed between variant allele carriers and non-carriers for *SCN4A* rs2302237 and the other analyzed polymorphisms (data not shown).

For PRO-CTCAE severity, results were generally consistent with those observed for FACT/GOG-Ntx scores (Additional file 1, Tables S2 and S3). In multivariate analyses, carriers of the *SCN4A* rs2302237 variant allele had a lower risk of moderate or greater symptom severity at 6 M. In contrast, carriers of the *SCN10A* rs6800541 variant allele tended to have a higher risk of moderate or greater symptom severity at 0 M.

**Table 4:** Association between FACT/GOG-Ntx scores and genetic factors

Variables		At treatment completion (0 M)	*p*-value	6 months after treatment completion (6 M)	*p*-value
*SCN4A* rs2302237	CC	15.45 ± 9.66	0.032	14.14 ± 10.05	0.014
	CT/TT	10.55 ± 6.60		8.36 ± 7.23	
*SCN5A* rs11720524	CC	12.73 ± 8.13	0.489	10.56 ± 9.66	0.470
	CG/GG	11.62 ± 8.54		10.57 ± 6.27	
*SCN9A* rs6746030	GG	12.6 ± 8.15	0.418	10.14 ± 8.36	0.354
	GA/AA	10.43 ± 9.09		14.71 ± 12.50	
*SCN9A* rs6754031	TT	13.32 ± 8.83	0.325	10.93 ± 8.20	0.402
	TG/GG	11.19 ± 7.20		10.06 ± 9.71	
*SCN10A* rs6800541	TT	11.59 ± 8.26	0.090	10.73 ± 9.28	0.869
	TC/CC	14.75 ± 7.77		10.10 ± 7.53	
*SCN10A* rs12632942	AA	11.74 ± 6.66	0.995	9.61 ± 7.73	0.825
	AG/GG	12.72 ± 8.83		10.98 ± 9.28	
*GSTP1* rs1695	AA	13.49 ± 8.30	0.008	11.79 ± 8.94	0.002
	AG/GG	7.23 ± 5.46		4.62 ± 5.16	

Data are presented as the mean ± standard deviation

Abbreviations: FACT/GOG-Ntx, Functional Assessment of Cancer Therapy/Gynecologic Oncology Group–Neurotoxicity

**Table 5:** Results of multiple linear regression analysis with the FACT/GOG-Ntx score as the dependent variable

	At treatment completion (0 M)				6 months after treatment completion (6 M)			
	B	β	95% CI	*p*-value	B	β	95% CI	*p*-value
(Constant)	9.979		1.674 to 18.285	0.019	6.074		−2.866 to 15.014	0.180
*SCN4A* rs2302237	−4.385	−0.261	−7.938 to − 0.831	0.016	−5.150	−0.286	−8.975 to − 1.326	0.009
*SCN5A* rs11720524	−1.722	−0.095	−5.631 to 2.186	0.382	−0.507	−0.026	−4.713 to 3.700	0.811
*SCN9A* rs6746030	−1.794	−0.064	−7.874 to 4.259	0.558	4.758	0.157	−1.786 to 11.302	0.151
*SCN9A* rs6754031	−2.816	−0.171	−6.374 to 0.742	0.119	−0.954	−0.054	−4.784 to 2.877	0.621
*SCN10A* rs6800541	4.564	0.247	0.393 to 8.735	0.032	0.121	0.006	−4.368 to 4.610	0.957
*SCN10A* rs12632942	2.774	0.156	−1.341 to 6.890	0.183	2.836	0.149	−1.594 to 7.266	0.206
*GSTP1* rs1695	−5.007	−0.231	−9.621 to − 0.394	0.034	−5.372	−0.231	−10.338 to − 0.406	0.034
Diabetes mellitus	5.501	0.195	−0.696 to 11.698	0.081	6.077	0.201	−0.593 to 12.747	0.073
Cumulative oxaliplatin dose, mg/m²	0.003	0.143	−0.002 to 0.008	0.179	0.005	0.195	0.000 to 0.010	0.069

The dependent variable was set as the FACT/GOG-Ntx score. Data are presented as the unstandardized regression coefficients (B), standardized regression coefficients (β), and 95% confidence intervals (CI)

Abbreviations: CI, confidence interval; FACT/GOG-Ntx, Functional Assessment of Cancer Therapy/Gynecologic Oncology Group–Neurotoxicity

## Discussion

In the present study, we investigated the association between *SCN* gene polymorphisms and chronic OXAIPN in Japanese patients with colorectal cancer using patient-reported outcomes as the principal assessment. Carriers of the *SCN4A* rs2302237 variant allele consistently exhibited lower FACT/GOG-Ntx scores than non-carriers at both 0 M and 6 M, indicating a lower patient-reported neuropathy-related symptom burden. In contrast, carriers of the *SCN10A* rs6800541 variant allele showed higher FACT/GOG-Ntx scores than non-carriers at 0 M, but the same association was not observed at 6 M. These findings suggest that individual *SCN* polymorphisms may show different relationships with OXAIPN depending on the SNP and the assessment time point.

SCN4A encodes Nav1.4, a skeletal muscle-type voltage-gated sodium channel that is predominantly expressed in skeletal muscle [20]. Because Nav1.4 is involved in the generation and propagation of action potentials at the neuromuscular interface, including the neuromuscular junction [21], functional variation in *SCN4A* may influence peripheral excitability and neuromuscular symptoms that overlap with patient-perceived neuropathy-related impairment. Regarding the association between OXAIPN and *SCN4A* polymorphisms, Argyriou et al. [16] reported that, in an overdominant model of *SCN4A* rs2302237 (CT vs. CC + TT), this polymorphism was associated with an increased incidence and severity of acute OXAIPN as well as a higher incidence of chronic OXAIPN. In contrast, Palugulla et al. [18] found no significant association between the *SCN4A* rs2302237 variant allele and the occurrence or severity of chronic OXAIPN. To our knowledge, the present finding that the dominant model of the *SCN4A* rs2302237 variant allele (CT + TT vs. CC) is associated with lower patient-reported chronic OXAIPN symptom burden has not been previously reported. Differences between the present findings and previous reports may partly be attributable to differences in genetic models, population background, and sample size, as well as differences in outcome assessment [22]. Most prior studies investigating associations between SCN polymorphisms and OXAIPN primarily relied on clinician-reported outcome measures, such as CTCAE grades or the Total Neuropathy Score. Although these measures are useful standardized assessments, they may not fully reflect the subjective burden of symptoms or their impact on daily functioning [23]. In chronic OXAIPN, symptom persistence and daily functional impairment are clinically important and can be captured sensitively by patient-reported outcome measures [24, 25]. Therefore, the use of FACT/GOG-Ntx and PRO-CTCAE in the present study may have influenced the detection of associations with patient-perceived neuropathy-related symptom burden. Because this study was exploratory, the present finding only suggests that *SCN4A* rs2302237 may be a candidate marker associated with patient-reported chronic OXAIPN symptom burden. Therefore, this association should be evaluated in larger independent validation cohorts in future studies.

Carriers of the *SCN10A* rs6800541 variant allele had significantly higher FACT/GOG-Ntx scores at 0 M, indicating greater neuropathy-related symptom burden at the end of oxaliplatin treatment. *SCN10A* encodes Nav1.8, which is highly expressed in peripheral sensory neurons and plays a critical role in neuronal excitability, pain transmission, and the pathophysiology of neuropathic pain in nociceptive pathways [26, 27]. These observations are consistent with prior findings suggesting that genetic variation in *SCN10A* may influence susceptibility to neuropathic symptoms [18]. However, because no significant association between *SCN10A* rs6800541 and FACT/GOG-Ntx scores was observed at 6 M, the relationship between this polymorphism and the persistence or recovery of symptoms after treatment completion remains unclear, and further validation in larger populations is required.

In addition, carriers of the *GSTP1* rs1695 variant allele had lower FACT/GOG-Ntx scores at both 0 M and 6 M than non-carriers. *GSTP1* rs1695 is a functional polymorphism that alters enzyme activity, and several studies have reported significant associations between this variant and oxaliplatin- or platinum-related neurotoxicity [8–10]. However, the existing evidence is inconsistent, as other studies have reported no significant relationship [28–30]. In a systematic review and meta-analysis of patients receiving platinum-based chemotherapy, *GSTP1* rs1695 was suggested to be associated with a reduced risk of gastrointestinal toxicity, whereas a direct protective effect against CIPN was not established [31]. Therefore, the reduced patient-reported symptom burden observed among *GSTP1* rs1695 variant allele carriers in the present study should be interpreted cautiously.

The distribution of variant allele frequencies in the present Japanese cohort also provides important background for interpreting the novelty and generalizability of this study. The frequency of the *SCN4A* rs2302237 T allele was relatively high in the present Japanese cohort (37.5%) and was broadly comparable to that reported in populations of European ancestry (36.9%) [32]. Because this SNP was associated with patient-reported OXAIPN outcomes in the present study, it may have clinical relevance in Japanese patients if the association is confirmed in future studies. In contrast, the allele frequencies of *SCN9A* rs6746030 A and *SCN10A* rs6800541 C were low in this cohort, which may have limited the statistical power to detect associations between these SNPs and OXAIPN. These considerations support the importance of evaluating *SCN* polymorphisms specifically in East Asian populations and of interpreting genetic association results in light of ethnic differences in allele frequency.

This study has several limitations. First, this was a relatively small exploratory study in which multiple genetic polymorphisms and outcomes were analyzed; therefore, false-positive findings due to multiple testing cannot be excluded. This study was designed as a hypothesis-generating investigation, and formal multiplicity correction was not applied because overly conservative correction in a small cohort may obscure potentially relevant candidate signals. Therefore, the present findings should not be interpreted as confirmatory evidence but should instead be considered cautiously in light of effect estimates and confidence intervals. Second, although the models were adjusted for diabetes mellitus and cumulative oxaliplatin dose, sex, anemia, alcohol use, and other potential confounders could not be fully incorporated because of the limited sample size. In addition, cumulative oxaliplatin dose is a treatment exposure determined during the treatment course and may reflect dose modification and tolerability. Third, the low frequency of some variant alleles, particularly *SCN9A* rs6746030, limited the statistical power to detect associations. Therefore, results related to low-frequency polymorphisms should be interpreted with caution.

## Conclusions

In this exploratory study of Japanese patients with colorectal cancer who received oxaliplatin-containing adjuvant chemotherapy, *SCN4A* rs2302237, *SCN10A* rs6800541, and *GSTP1* rs1695 showed associations with patient-reported chronic OXAIPN outcomes. Among these polymorphisms, *SCN4A* rs2302237 was consistently associated with lower FACT/GOG-Ntx scores at treatment completion (0 M) and 6 months after treatment completion (6 M), suggesting a lower patient-reported neuropathy-related symptom burden. In contrast, *SCN10A* rs6800541 was associated with higher FACT/GOG-Ntx scores at treatment completion (0 M), whereas no similar association was observed 6 months after treatment completion (6 M). These results suggest that different polymorphisms may be related to different aspects of OXAIPN assessment over time. The present findings suggest that *SCN4A* rs2302237 may be a candidate marker associated with patient-reported chronic OXAIPN symptom burden in Japanese patients; however, this finding is based on an exploratory analysis, and the clinical utility of *SCN4A* genotyping has not yet been established. Therefore, larger validation studies are needed.

## Supplementary Information

Below is the link to the electronic supplementary material.


Supplementary Material 1: Additional file 1: Supplementary tables for analyses of genetic polymorphisms and chronic oxaliplatin-induced peripheral neuropathy. This supplementary file contains Tables S1–S3. Table S1 presents exploratory comparisons of clinical background factors, including age, sex, BMI, cumulative oxaliplatin dose, and number of oxaliplatin-containing cycles, between SCN4A rs2302237 variant allele carriers and non-carriers. Tables S2 and S3 present detailed results of analyses evaluating associations between genetic polymorphisms and chronic oxaliplatin-induced peripheral neuropathy assessed using PRO-CTCAE outcomes. Table S1. Comparison of clinical background factors between SCN4A rs2302237 variant allele carriers and non-carriers Table S2. Association between PRO-CTCAE symptom severity (≥ moderate) and genetic polymorphismsTable S3. Logistic regression analysis for the occurrence of PRO-CTCAE symptom severity ≥ moderate


## Data Availability

The datasets generated and/or analyzed during the current study are available from the corresponding author on reasonable request.

## Abbreviations

BMI: Body mass index
CIPN: Chemotherapy-induced peripheral neuropathy
FACT/GOG-Ntx: Functional assessment of cancer therapy/Gynecologic oncology group–Neurotoxicity
HWE: Hardy–weinberg equilibrium
Nav: Voltage-gated sodium channel α-subunit
OXAIPN: Oxaliplatin-induced peripheral neuropathy
PRO-CTCAE: Patient-reported outcomes version of the common terminology criteria for adverse events
QOL: Quality of life
SCN: Voltage-gated sodium channel
SNP: Single-nucleotide polymorphism
