# Supplementary material for: Association between voltage-gated sodium channel gene polymorphisms and chronic oxaliplatin-induced peripheral neuropathy in Japanese patients with colorectal cancer
Source: J Pharm Health Care Sci. 2026 Jun 19;12:80. doi: 10.1186/s40780-026-00601-2 (PMC13536608; doi:10.1186/s40780-026-00601-2)
Supplement: Supplementary file 1 [file 40780_2026_601_MOESM1_ESM.docx]

Table S1. Comparison of clinical background factors according to *SCN4A* rs2302237 genotype

| Variable | CC genotype (n = 29) | CT/TT genotypes (n = 47) | p-value |
| --- | --- | --- | --- |
| Age, years | 65.9 ± 10.6 | 63.8 ± 9.6 | 0.199 |
| Sex, male | 21 (72) | 29 (62) | 0.456 |
| BMI, kg/m² | 22.2 ± 2.8 | 22.7 ± 4.2 | 0.906 |
| Diabetes mellitus | 2 (7) | 5 (11) | 0.702 |
| Cumulative oxaliplatin dose, mg/m² | 808.2 ± 196.9 | 782.1 ± 178.4 | 0.406 |
| Number of oxaliplatin-containing cycles | 8 (7–8) | 8 (7–8) | 0.631 |

Data are presented as mean ± standard deviation, median (interquartile range), or number (percentage), as appropriate.

Abbreviations: BMI, body mass index; IQR, interquartile range.

Table S2. Association between classification as PRO-CTCAE “severity” ≥ moderate and genetic factors

| Variables | | At treatment completion (0M) | | p-value | 6 months after treatment completion (6M) | | p-value |
| --- | --- | --- | --- | --- | --- | --- | --- |
|  |  | None–Mild | Moderate–Very severe |  | None–Mild | Moderate–Very severe |  |
| *SCN4A* rs2302237 | CC | 9 (31.0) | 20 (69.0) | 0.410 | 13 (44.8) | 16 (55.2) | 0.016 |
|  | CT/TT | 19 (40.4) | 28 (59.6) |  | 34 (72.3) | 13 (27.7) |  |
| *SCN5A* rs11720524 | CC | 18 (32.7) | 37 (67.3) | 0.229 | 33 (60.0) | 22 (40.0) | 0.593 |
|  | CG/GG | 10 (47.6) | 11 (52.4) |  | 14 (66.7) | 7 (33.3) |  |
| *SCN9A* rs6746030 | GG | 26 (37.7) | 43 (62.3) | 1.000 | 44 (63.8) | 25 (36.2) | 0.417 |
|  | GA/AA | 2 (28.6) | 5 (71.4) |  | 3 (42.9) | 4 (57.1) |  |
| *SCN9A* rs6754031 | TT | 15 (34.1) | 29 (65.9) | 0.560 | 30 (68.2) | 14 (31.8) | 0.182 |
|  | TG/GG | 13 (40.6) | 19 (59.4) |  | 17 (53.1) | 15 (46.9) |  |
| *SCN10A* rs6800541 | TT | 24 (42.9) | 32 (57.1) | 0.105 | 36 (64.3) | 20 (35.7) | 0.463 |
|  | TC/CC | 4 (20.0) | 16 (80.0) |  | 11 (55.0) | 9 (45.0) |  |
| *SCN10A* rs12632942 | AA | 8 (34.8) | 15 (65.2) | 0.806 | 15 (65.2) | 8 (34.8) | 0.690 |
|  | AG/GG | 20 (37.7) | 33 (62.3) |  | 32 (60.4) | 21 (39.6) |  |
| *GSTP1* rs1695 | AA | 19 (30.2) | 44 (69.8) | 0.012 | 36 (57.1) | 27 (42.9) | 0.114 |
|  | AG/GG | 9 (69.2) | 4 (30.8) |  | 11 (84.6) | 2 (15.4) |  |

Data are presented as the number (percentage).

Abbreviations: PRO-CTCAE, Patient-Reported Outcomes version of the Common Terminology Criteria for Adverse Events.

Table S3. Logistic regression analysis for classification as PRO-CTCAE “severity” ≥ moderate

|  | At treatment completion (0M) | | | | 6 months after treatment completion (6M) | | | |
| --- | --- | --- | --- | --- | --- | --- | --- | --- |
| Explanatory variable | B | OR | 95% CI | p-value | B | OR | 95% CI | p-value |
| (Constant) | 1.559 | 4.752 |  | 0.351 | -3.184 | 0.041 |  | 0.071 |
| *SCN4A* rs2302237 | -0.372 | 0.689 | 0.230 to 2.069 | 0.507 | -1.283 | 0.277 | 0.088 to 0.871 | 0.028 |
| *SCN5A* rs11720524 | -1.003 | 0.367 | 0.113 to 1.191 | 0.095 | -0.534 | 0.586 | 0.162 to 2.122 | 0.416 |
| *SCN9A* rs6746030 | 0.546 | 1.727 | 0.227 to 13.141 | 0.598 | 1.001 | 2.721 | 0.451 to 16.419 | 0.275 |
| *SCN9A* rs6754031 | -0.764 | 0.466 | 0.155 to 1.403 | 0.174 | 0.663 | 1.941 | 0.633 to 5.945 | 0.246 |
| *SCN10A* rs6800541 | 1.508 | 4.519 | 1.056 to 19.333 | 0.042 | 0.369 | 1.447 | 0.407 to 5.141 | 0.568 |
| *SCN10A* rs12632942 | 0.299 | 1.348 | 0.379 to 4.795 | 0.645 | 0.667 | 1.948 | 0.495 to 7.666 | 0.340 |
| *GSTP1* rs1695 | -1.848 | 0.158 | 0.038 to 0.657 | 0.011 | -1.261 | 0.283 | 0.049 to 1.628 | 0.157 |
| Diabetes mellitus | -0.436 | 0.647 | 0.100 to 4.201 | 0.648 | -0.659 | 0.517 | 0.103 to 2.610 | 0.425 |
| Cumulative oxaliplatin dose, mg/m^2^ | 0.000 | 1.000 | 0.997 to 1.003 | 0.979 | 0.004 | 1.004 | 1.001 to 1.007 | 0.012 |

The dependent variable was set as the occurrence of PRO-CTCAE symptom severity ≥ moderate. Data are presented as the regression coefficient (B), odds ratio (OR), and 95% confidence interval (CI).

Abbreviations: CI, confidence interval; OR, odds ratio; PRO-CTCAE, Patient-Reported Outcomes version of the Common Terminology Criteria for Adverse Events.
